# Detecting joint inflammation by an LED-based photoacoustic imaging system: a feasibility study

**DOI:** 10.1117/1.JBO.23.11.110501

**Published:** 2018-11-29

**Authors:** Janggun Jo, Guan Xu, Yunhao Zhu, Mary Burton, Jeffrey Sarazin, Elena Schiopu, Girish Gandikota, Xueding Wang

**Affiliations:** a University of Michigan, Department of Biomedical Engineering, Ann Arbor, Michigan, United States; b University of Michigan Medical School, Department of Radiology, Ann Arbor, Michigan, United States; c University of Michigan Medical School, Division of Rheumatology, Department of Internal Medicine, Ann Arbor, Michigan, United States

**Keywords:** photoacoustic imaging, light-emitting diode, inflammatory arthritis, joint

## Abstract

Light-emitting diode (LED) light sources have recently been introduced to photoacoustic imaging (PAI). The LEDs enable a smaller footprint for PAI systems when compared to laser sources, thereby improving system portability and allowing for improved access. An LED-based PAI system has been employed to identify inflammatory arthritis in human hand joints. B-mode ultrasound (US), Doppler, and PAIs were obtained from 12 joints with clinically active arthritis, five joints with subclinically active arthritis, and 12 normal joints. The quantitative assessment of hyperemia in joints by PAI demonstrated statistically significant differences among the three conditions. The imaging results from the subclinically active arthritis joints also suggested that the LED-based PAI has a higher sensitivity to angiogenic microvascularity compared to US Doppler imaging. This initial clinical study on arthritis patients validates that PAI can be a potential imaging modality for the diagnosis of inflammatory arthritis.

Inflammatory arthritis caused by autoimmune disorders is a chronic, progressive set of diseases with worldwide prevalence.^1^ Rheumatoid arthritis (RA), one such type of inflammatory arthritis, has symptoms of stiffness, pain, and swelling of the joints. In addition, RA synovium shows hypoxia, neoangiogenesis, and synovial proliferation within the peripheral joints.^2^^–^^4^ Synovial angiogenesis is an important feature in the early stage of development and perpetuation of inflammatory arthritis. Along with magnetic resonance imaging (MRI), ultrasound (US) Doppler imaging has been employed as the main imaging modality in identifying increased vascularity related to inflammatory arthritis.^5^^,^^6^ The use of MRI is not widespread for general clinical screening and diagnosis due to the high cost and limited accessibility. US Doppler imaging can offer high-resolution images of joint structures and has proven sensitivity in detecting blood flow. US Doppler imaging, however, is more sensitive to the fast blood flow in relatively large vessels. Slow blood flow in smaller capillaries, which are more clinically and pathologically relevant to early active synovitis,^7^ could be missed by US Doppler imaging.

Recently, photoacoustic imaging (PAI) has also shown the capability of identifying active synovitis in human finger joints.^8^^,^^9^ With the unique capability of mapping highly sensitive optical information in deep tissue with excellent spatial resolution,^10^ this emerging imaging technique has been developed and investigated for various preclinical and clinical applications.^11^^–^^13^ Presenting endogenous optical absorption contrast in tissues, PAI, when combined with B-mode US, can provide additional functional and molecular information such as blood volume and blood oxygen saturation which are highly valuable in diagnosis of many pathological conditions.^14^^–^^17^ A previous study in our laboratory demonstrated the potential of PAI for diagnosis of inflammatory arthritis based on the detection of hyperemia (i.e., increased blood content) and hypoxia (i.e., decreased blood oxygen saturation) in the affected joints.^8^ The intense hyperemia in the affected joint is not only from the hypervascularization but also the dilatation of veins and capillaries and can be detected well by PAI due to its high sensitivity to hemoglobin optical absorption. Because of the increased metabolic demand of the inflamed synovium and the relatively inadequate oxygen delivery of the inflamed joint, the inflamed synovium shows profound hypoxia which can also be detected by multiwavelength PAI based on the spectroscopic difference between oxygenated and deoxygenated hemoglobin.

PAI has mostly used solid-state pulsed lasers such as Q-switched Nd:YAG lasers as light sources. These laser systems, with output energy of several to hundreds mJ at each pulse, provide desirable signal-to-noise ratios (SNR). Although these lasers work fine for PAI systems developed or used in laboratories, their high cost, large footprint, and lesser mobility could substantially hinder the translation of PAI technology from bench to clinic. Using low-cost and small-size light source such as light-emitting diode (LED) and xenon flash lamp as the alternative illumination source for PAI has many advantages^18^^–^^21^ and may fundamentally remove the cost and practicality barriers of this fast-developing biomedical imaging technology.

In this work, a PAI system using LED arrays as the light source was introduced into the clinical study of inflammatory arthritis. The LED PAI was employed in assessing peripheral joints from arthritis patients and normal volunteers. The images from clinically active arthritis, subclinically active arthritis, and normal groups were compared and statistically analyzed. PAI results were also compared with those from US Doppler imaging to explore the potential advantages of this imaging technology over existing modalities.

The LED PAI system used in this study was built by Prexion Corporation (AcousticX, Tokyo, Japan), as shown in Fig. 1. The details of this system, including the safety for applications on human subjects, have been introduced in our previous publication.^20^ When imaging a human hand joint, two LED arrays were placed at the two sides of an US probe with 45 deg incline to provide a total energy of 400  μJ per pulse at 850-nm wavelength. The pulse duration and the repetition rate of the light pulse were 35 ns and 4 kHz, respectively. This system enables photoacoustic (PA) and US dual-modality imaging using either a 7-MHz linear probe or a 10-MHz linear probe. When working with the 7-MHz probe (128 elements), as employed in this study, this system offers spatial resolution of 310  μm lateral and 250  μm axial, and an image depth up to 30 mm for PAI. The PA signals were acquired at each LED pulse and averaged 384 times leading to a frame rate of ∼10  Hz. After processing the PA signals using frequency band pass filter, log compression, and time gain compensation, the PA images were reconstructed by the AcousticX system using the delay-and-sum method, and then displayed after applying dynamic range control which enhances high intensity signals. When acquiring PA images from human subjects, a consistent gain of 56 was employed so that the comparison among different groups can be made.

**Fig. 1:**
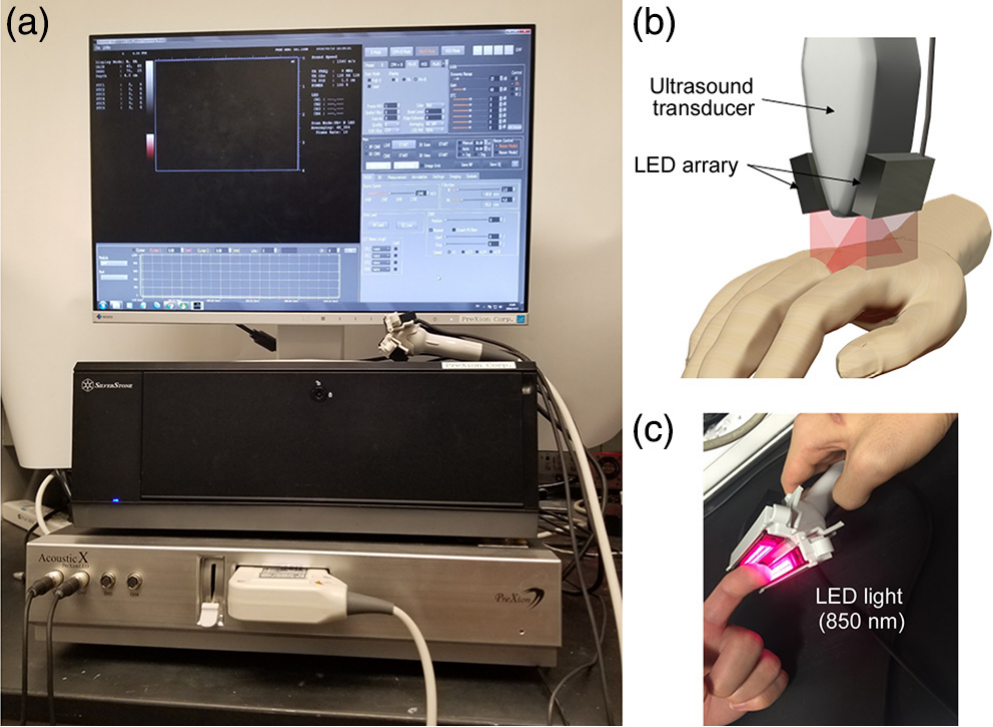
LED-based PAI system. (a) Photograph of the imaging system. (b) The LED array light source located on both sides of the US transducer illuminates 850-nm wavelength of light to scan human hand joints. (c) Photograph of the probe.

All procedures for human subjects in this study were approved by the Institutional Review Board (IRB) of the University of Michigan Medical School. In this study, 17 inflammatory arthritis patients and 12 healthy volunteers (both men and women, over 18 years old) were provided written informed consent and participated. The arthritis patients had apparent swelling and pain in at least one of their finger joints on clinical exam. Board-certified rheumatologists at the University of Michigan Medical School identified the affected finger joints following the American College of Rheumatology (ACR) criteria. Healthy volunteers who did not have symptoms and no clinical record of inflammatory arthritis were also recruited.

The patients had an US Doppler scan of the hands and wrists joints during their clinic visits. In addition, the diseased conditions were also confirmed by the Doppler mode of a commercial US unit (Z.ONE PRO, ZONARE, Mountain View, California) with a linear probe (L14-5W, ZONARE) right before the PAI of the affected joints. The Doppler function was configured with a pulse repetition frequency of 1500 Hz and a color scale of 7.5 to −7.5  cm/s. The joint was scanned along the sagittal plane. The imaging plane where active flow was found in the synovium by the Doppler US was later revisited by the LED PAI. The patients’ joints that did not show prominent flow in the US Doppler imaging were also scanned later by the LED PAI in search of increased vasculatures. During the PAI, the patient’s hand was placed in the warm water (37°C) for US coupling. The total scanning time including both Doppler US and LED PAI of each joint was less than 10 min.

As an example of clinically active arthritis case, Fig. 2 shows Doppler US B-scan images [Figs. 2(b) and 2(c)] and the parallel PAI image [Fig. 2(d)] of a human metacarpophalangeal (MCP) joint with inflammation. Figure 2(b) was obtained by a sonographer during a clinic visit. Figure 2(c) is the US Doppler image obtained right before the PAI scan. The US images in Figs. 2(b) and 2(d) show highly correlated MCP joint structures, as marked by the yellow arrows in the images. The hyperemia displayed by colored Doppler images in Figs. 2(b) and 2(c) can also be found at the same position in the superimposed PA and US image [Fig. 2(d)], where the pseudocolor red pixels were blood signals detected by PAI.

**Fig. 2:**
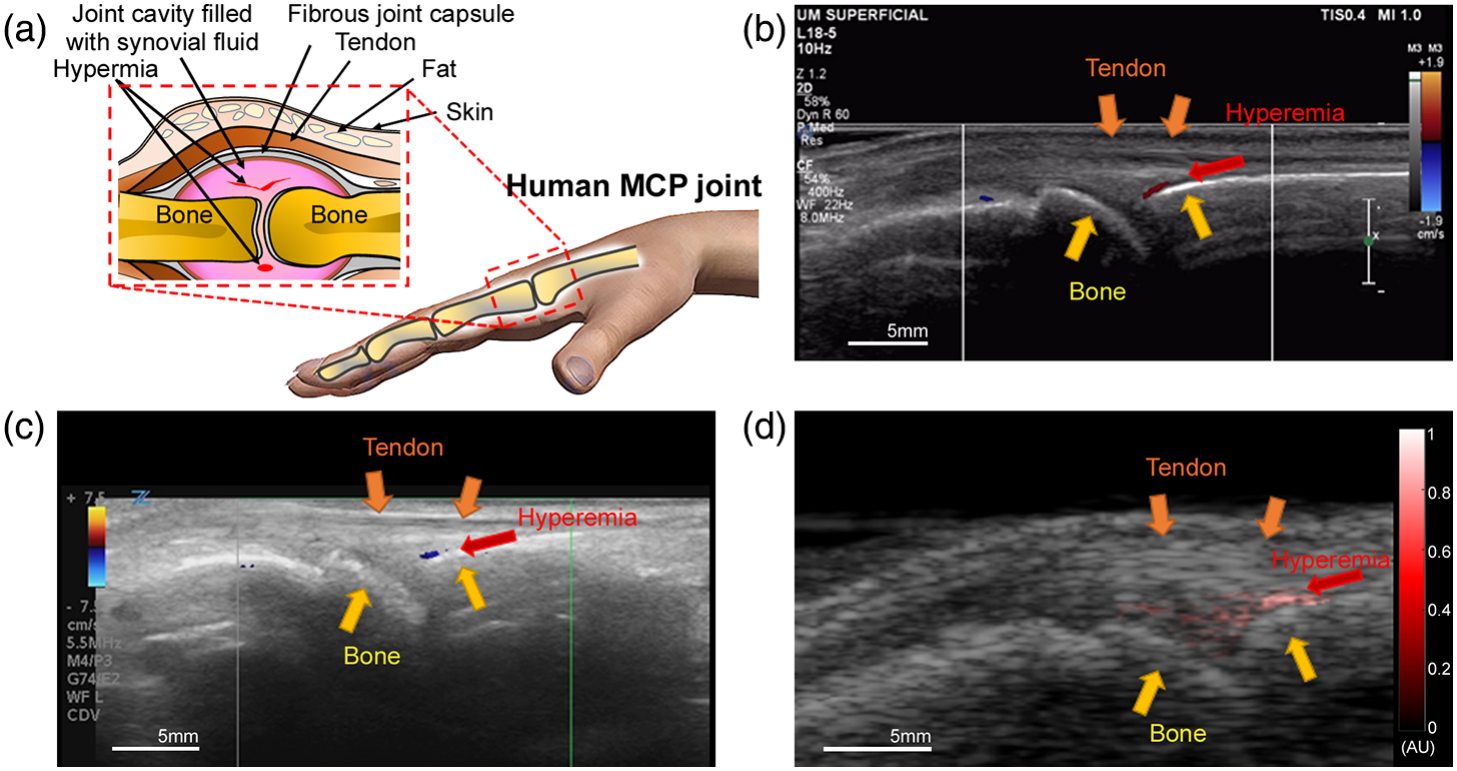
Scanning an inflammatory human MCP joint with US Doppler imaging and LED PAI. (a) Sketch of an inflamed human MCP joint. (b) US Doppler image of the MCP joint obtained during the clinic visit, showing hyperemia in synovium. (c) US Doppler image of the same joint obtained right before PAI scan, indicating hyperemia in the same position. (d) PA and B-mode US combined image of the same joint, where the red pixels in the pseudocolor PA image indicate hyperemia in the same position in the joint.

We also observed cases where hyperemia can be recognized in PA images but not by Doppler US imaging including both US Doppler imaging during the clinic visit and US Doppler imaging using the ZONARE system right before the PAI scan. Same as the clinically active arthritis patients, this group of patients also had swelling and pain in affected finger joints, as confirmed by the board-certified rheumatologists following the ACR criteria. However, the activity in the affected joints was not strong enough to be detected by the US Doppler imaging systems used. These patients, with hyperemia seen only on PAI images, were categorized as a separate group which was defined as subclinically active arthritis.

Following a similar procedure, each healthy volunteer also had an US Doppler scan, followed by a subsequent scan of the same finger joints using the LED PAI. Unlike the results from the arthritic joints, no prominent hyperemia can be identified in the synovium of the normal joints, which was confirmed by both US Doppler imaging and PAI.

Figure 3 shows the representative US Doppler (left) and PA (right) images of the three groups compared in this study, including clinically active arthritis joints, subclinically active arthritis joints, and normal healthy joints. Figure 3(a) shows a clinically active arthritis case with hyperemia seen at the same location in both Doppler US and PA images. Figure 3(b) shows a subclinically active arthritis case in which hyperemia was only seen in PA image but not in US Doppler image of the patient’s MCP joints. Figure 3(c) shows a normal case where no hyperemia can be seen in either US Doppler or PA images.

**Fig. 3:**
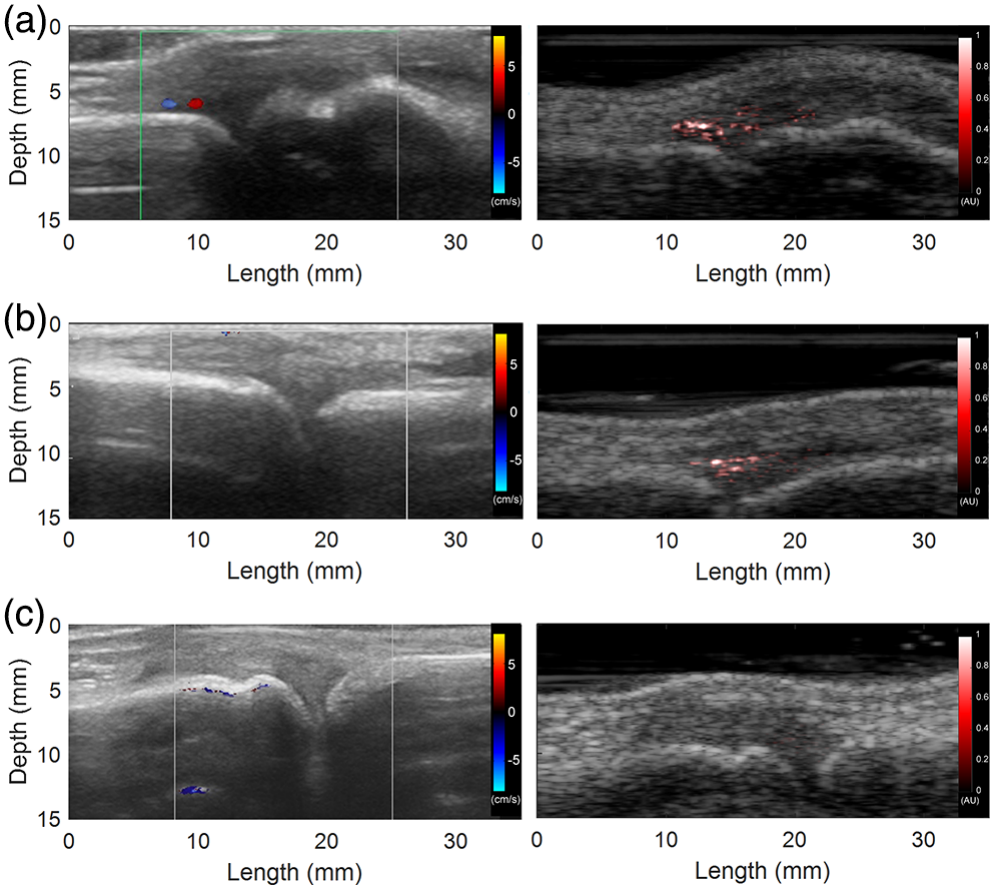
(Left) US Doppler images and (right) PA images of human MCP joints. (a) The images of a clinically active inflammatory arthritis joint showing hyperemia in both US Doppler and PA images. (b) The images of a subclinically active inflammatory arthritis joint showing hyperemia in PA image only but not in US Doppler image. (c) The images of a normal joint showing no hyperemia in either US Doppler image or PA image.

To characterize the capability of PAI utilizing LED light source in differentiating the three groups studied [i.e., clinically active arthritis group (n=12), subclinically active arthritis group (n=5), and normal group (n=12)], the imaging results from the three groups were compared. With the pseudocolor PA images of each joint acquired, we evaluated the hyperemia as a biomarker of joint inflammation by quantifying two parameters, including (1) the density of colored pixels and (2) the average intensity of colored pixels in the joint area. The detailed methods quantified these two parameters were described in our previous publication.^8^ Briefly, for each pseudocolor PA image of a joint, the density of colored pixels was calculated by dividing the number of colored pixels by the number of total pixels in the joint area; the average intensity of colored pixels was calculated by the sum of the intensities of all colored pixels divided by the number of colored pixels in the joint area.

The quantified parameters of the three groups are compared in Fig. 4. Figure 4(a) shows the box plots of the density of colored pixels in the joint area. The averages and the standard deviations of the three groups are 14.0±5.80 (%), 7.0±3.20 (%), and 0.4±0.51 (%), respectively. To examine whether there is statistically significant difference in this first parameter between any of the two groups, two tailed t-test was performed using the built-in functions of the MATLAB (R2016b, Mathworks, Natick, Massachusetts). The statistical analyses show that any of the two groups can be differentiated by PAI based on the quantified density of colored pixels in the joint area. The p values from the two-tailed t-tests were 0.024 for differentiating the clinically active group and the subclinically negative group, $5.6\times 10^{-8}$ for differentiating the clinically active group and the normal group, and $3.1\times 10^{-6}$ for differentiating the subclinically active group and the normal group.

**Fig. 4:**
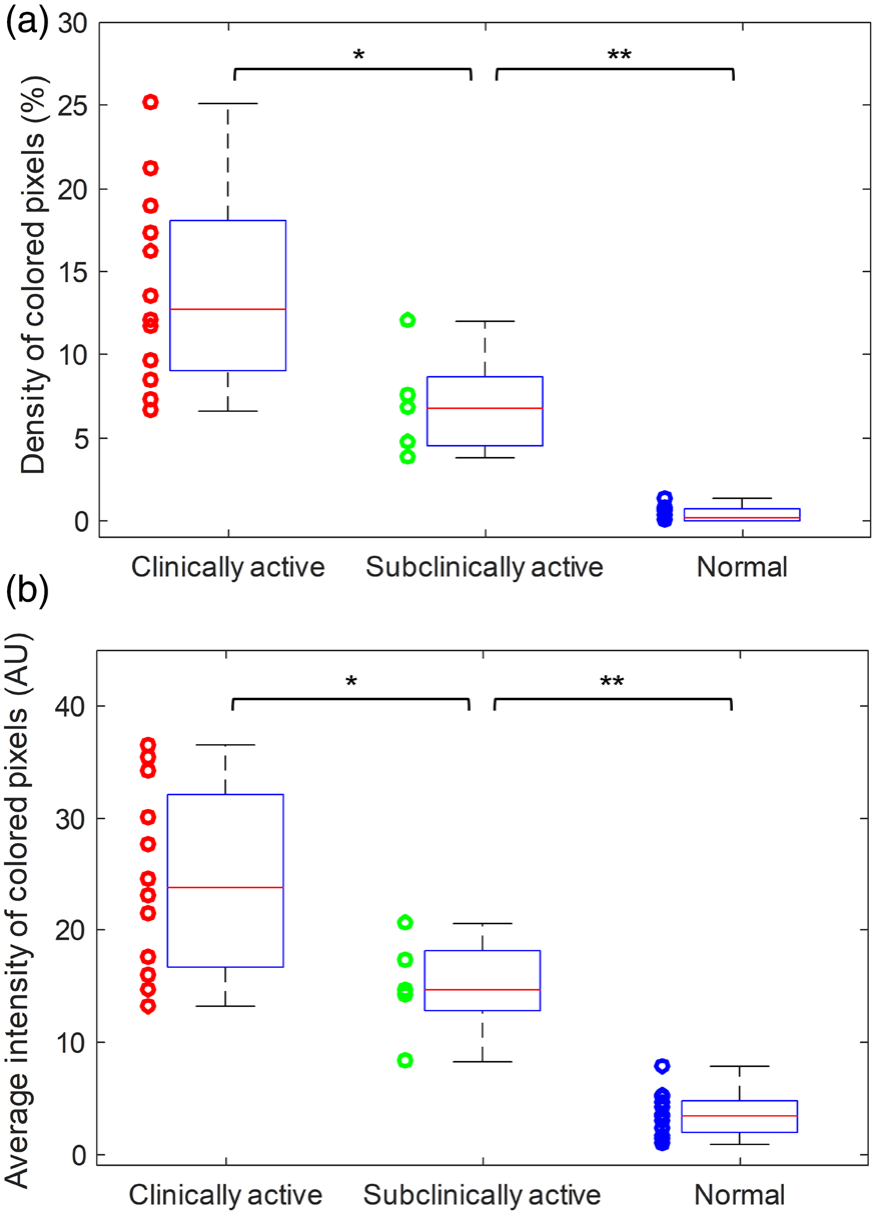
Statistical studies comparing the hyperemia in the three groups of joints (i.e., clinically active arthritis, n=12; subclinically active arthritis, n=5; and normal, n=12) as quantified by LED PAI. (a) The quantified results showing the density of colored pixels in pseudocolor PA images of the three groups. (b) The quantified results showing the average intensity of colored pixels in pseudocolor PA images of the three groups. Note: * is for p<0.05, and ** is for p<0.005.

Figure 4(b) shows the box plots of the average intensity of colored pixels in the joint area for the three groups. The averages and the standard deviations of the three groups are 24.53±8.28, 15.03±4.54, and 3.56±1.97, respectively. Similarly, two-tailed t-test was performed to examine whether there is statistically significant difference in this second parameter between any of the two groups. The statistical analyses show that any of the two groups can be differentiated by PAI based on the quantified average intensity of colored pixels in the joint area. The p values from the two-tailed t-tests were 0.030 for differentiating the clinically active group and the subclinically negative group, $2.0\times 10^{-8}$ for differentiating the clinically active group and the normal group, and $2.0\times 10^{-6}$ for differentiating the subclinically active group and the normal group.

Considering that both the clinically active group and the subclinically active group are joints with inflammation, we also examined whether there is statistically significant difference between the PA images from the arthritic joints (including both clinically active and subclinically active joints) and the normal joints. Two-tailed t-test was conducted for each of the two quantified parameters (i.e., the density of colored pixels and the average intensity of colored pixels), and p values of $5.5\times 10^{-7}$ and $8.8\times 10^{8}$ were achieved, respectively, demonstrating that LED PAI is capable of differentiating arthritic joints from the normal joints.

This initial study on arthritis patients and normal volunteers demonstrated that the LED-based PAI can detect the earliest functional changes of inflammation in human peripheral joints. In addition to the structural details and blood flow appreciated by the pulse-echo and Doppler US, the PAI provides unique information regarding subtle changes in blood content independent of flow. The improved sensitivity to blood content brought by the PAI facilitates the differentiation between mild inflammatory joints and the healthy control cases, where the US Doppler may not be able to distinguish. The quantitative PA measurements have also demonstrated narrower and sharper criteria for identifying neovascularity in synovium. In addition, the LED light source enables an imaging system with a small footprint and excellent portability, benefiting clinical translation and commercialization.

To achieve sufficient SNR when working with the weak pulse energy (400  μJ) from the two LED arrays, PA signals from the joint were averaged extensively over 384 pulses which improved the SNR by $\sqrt{384}\approx 20$ times. This SNR is comparable to that produced by a single pulse with a pulse energy of 400  μJ×20=8  mJ from a class-IV laser without signal averaging.^20^ When maintaining the same illumination power of 400  μJ×4  KHz=1.6  W, a class-IV laser with pulse energy of 160 mJ and a pulse repetition of 10 Hz (for the same imaging frame rate) can lead to an SNR which is 160  mJ/8  mJ=20 times higher. Although this is one of the limitations of the LED-based PAI systems compared to those based on powerful class-IV lasers, the SNR as well as the imaging depth achieved in this study was sufficient for detecting the inflammation in human peripheral joints.

In future studies, we will explore the feasibility of LED PAI in evaluating the decreased blood oxygen saturation, i.e., hypoxia, in the arthritic joints as another imaging biomarker of synovitis. To achieve that, dual-color LED arrays providing light at two different wavelengths will be employed.^20^ In addition, three-dimensional (3-D) imaging of the volumetric information of tissue inflammation is also under investigation. With a cost in imaging speed, 3-D PAI could be more sensitive and more quantitative in assessing the functional changes associated with progression of arthritis and response to treatment. Although our initial study was focused on the peripheral joints of human hands and feet, the 30-mm imaging depth offered by the LED PAI system may be useful in the study of larger human joints such as ankle and wrist which are also affected often by inflammatory arthritis.
