# Detecting joint inflammation by an LED-based photoacoustic imaging system: a feasibility study (Erratum)

**DOI:** 10.1117/1.JBO.26.5.059802

**Published:** 2021-05-18

**Authors:** Janggun Jo, Guan Xu, Yunhao Zhu, Mary Burton, Jeffrey Sarazin, Elena Schiopu, Girish Gandikota, Xueding Wang

**Affiliations:** a University of Michigan, Department of Biomedical Engineering, Ann Arbor, Michigan, United States; b University of Michigan Medical School, Department of Radiology, Ann Arbor, Michigan, United States; c University of Michigan Medical School, Division of Rheumatology, Department of Internal Medicine, Ann Arbor, Michigan, United States

## Abstract

An error in the first author’s name is corrected.

This article [*J. Biomed. Opt.* **23**(11), 110501 (2018) doi: 10.1117/1.JBO.23.11.110501 was originally published on 29 November 2018 with an error in the name of the first author. Janggun Jo was misspelled as “Junggun Jo.” The error was corrected on 7 May 2021.

